# Transitional care gaps and caregiver burden after ICU discharge: a cross-sectional study of patient–caregiver dyads

**DOI:** 10.3389/fpubh.2026.1915395

**Published:** 2026-08-28

**Authors:** Hongfei Wang, Ning Luo, Yin Li

**Affiliations:** Department of Intensive Care Unit, Tianjin First Central Hospital, Tianjin, China

**Keywords:** caregiver burden, critical illness, family caregivers, post-intensive care syndrome, rural health disparities, transitional care

## Abstract

**Background:**

Survivors of critical illness experience post-intensive care syndrome (PICS), and family caregivers bear a parallel burden, PICS-family, while gaps in transitional-care services persist. We aimed to determine the prevalence of a high transitional-care gap after intensive care unit (ICU) discharge (primary outcome) and to assess whether it was associated with caregiving burden and psychological strain; a secondary aim was to assess residence-related access barriers.

**Methods:**

A single-center cross-sectional study of patient–caregiver dyads at Tianjin First Central Hospital, China, from February 2022 to March 2026, using consecutive sampling (2,270 of 2,981 eligible dyads; 76.1%). Eligible dyads comprised a patient aged ≥18 years with an ICU stay ≥24 h who survived to hospital discharge and an unpaid primary family caregiver. Caregivers were assessed once, at a mean of 21.3 (SD 4.4) days after ICU discharge, using a researcher-developed eight-domain battery. The primary outcome was a high transitional-care gap (≥5 unmet services of 15). Rehospitalization or emergency department (ED) visit was defined as unplanned readmission or ED attendance between hospital discharge and assessment. Analyses were conducted in R version 4.3.2 using chi-square tests, Spearman correlation, and multivariable linear and logistic regression.

**Results:**

All measures were completed by the family caregiver, reporting on both dyad members. Among 2,270 dyads (patient age 57.4 ± 14.4 years; 43.6% women; 65.3% mechanically ventilated), patient-level outcomes were rehospitalization/ED visit in 576 (25.4%) and mean vulnerability 28.5 ± 5.3; caregiver-level outcomes were a high transitional-care gap in 1,710 (75.3%; 95% CI 73.5–77.1), high burden in 761 (33.5%), and mean strain 27.5 ± 4.1. Unmet need was highest for psychological support of the caregiver (76.8%) and patient (74.2%). A high gap was not associated with greater burden: 22.1 ± 7.7 with a high gap versus 23.1 ± 7.8 without (SMD ≤ 0.14), and the unmet-need score did not predict burden after adjustment (*β* = 0.00, 95% CI − 0.02 to 0.01, *p* = 0.69). Burden was predicted by caregiving hours/day (*β* = 0.83, 95% CI 0.81–0.85, *p* < 0.001; adjusted *R*^2^ = 0.876), and strain by burden (*β* = 0.73) and patient vulnerability (*β* = 0.25; both *p* < 0.001). Furthermore, rural caregivers reported more of the 13/14 access barriers (high barrier burden 52.5% vs. 26.9%; prevalence ratio 1.95, *p* < 0.001) without higher gaps, burden, or rehospitalization (all *p* > 0.19).

**Conclusion:**

Three-quarters of dyads left the ICU with a high transitional-care gap, concentrated in psychological support. However, the gap was not associated with caregiver burden, which was measured by caregiving intensity and patient dependency. Service expansion and caregiver support are therefore distinct targets, and neither measure identifies the families needing the other.

## 1 Introduction

Survival from critical illness has improved, but recovery does not end at intensive care unit (ICU) discharge. A growing share of patients leave with persistent physical, cognitive, or psychological impairment, collectively termed post-intensive care syndrome (PICS) (1–3). The weeks that follow are a heightened window of vulnerability, during which patients and caregivers transition from continuous monitoring to a community setting with comparatively little structured support (4, 5). Qualitative work with survivors and families transitioning home describes a recurring pattern: patients feel discharged without a plan, caregivers face exhaustion without a safety net, and both manage emergent needs with inadequate guidance (5, 6). Conceptual models of post-ICU recovery therefore call for systematic reconciliation of pre-ICU and current status at every care transition, and for coordinated engagement between critical care and community providers across the recovery continuum (1–3).

That transition is rarely resourced. A binational survey of 83 ICUs found that only 29% offered any follow-up service for patients and 25% for caregivers, with funding and staffing cited as the principal constraints (7). A scoping review of non-clinic-based interventions identified only six of 45 eligible studies directed at caregivers, most evaluating feasibility rather than effectiveness (8, 9). Where structured follow-up has been adequately resourced, it works: nurse-led telephone follow-up and post-discharge transitional-care programs have reduced readmissions and improved symptom control (10, 11), and tele-delivered psychotherapeutic support for ICU families has produced large reductions in distress, anxiety, and depression (12, 13). Access is nonetheless unevenly distributed in China, with capacity lowest in economically underdeveloped and predominantly rural regions (14), where patients with chronic disease nonetheless report willingness to use telemedicine in place of in-person care (15).

What the system does not provide, families absorb. The companion construct PICS-family (PICS-F) describes the depression, anxiety, post-traumatic stress, and fatigue caregivers develop while supporting patients through recovery. At 3 months, rates were 22.3% for anxiety or depressive symptoms and 26.6% for post-traumatic stress, with admission-day distress and low perceived social support stronger predictors than any feature of the illness course (3, 16). Chinese dyads assessed 2 weeks after discharge showed moderate-to-severe burden in nearly 40% of caregivers, with rural residence, younger caregiver age, and round-the-clock caregiving among the independent risk factors (17), and at 5 years, job loss and reduced working hours predicted caregiver anxiety and depression, indicating that economic disruption is central to caregiving burden rather than incidental to it (18). This burden is prognostic rather than merely descriptive: each point on the Zarit Burden Interview raised the odds of a 30-day emergency department revisit (19). The demand is also durable and anchored in the patient’s condition rather than the caregiver’s. Among caregivers in long-term acute care, nearly 80% identified going home as the central goal, yet only 38% of patients had achieved it within a year (20), and one-year outcome data on chronic critical illness show 64% of survivors retaining moderate-to-severe disability and only 62% independent in personal care (21).

Therefore, two questions remain unanswered, and both concern the dyad rather than either member alone. Existing instruments were designed to quantify the caregiver’s psychological state rather than the availability of services. The Preparedness for Caregiving Scale demonstrates strong internal consistency among caregivers of patients with chronic illness, and the Zarit Burden Interview remains the most widely used measure of burden (22, 23). Yet neither was developed for the post-ICU transition, and none captures unmet service need as distinct from psychological burden. Because patients and caregivers are typically studied separately, it is unknown whether service deprivation and caregiver strain identify the same families, or whether documented inequities in healthcare capacity translate into measurable inequities in post-ICU transitional-care access. We therefore conducted a single-center cross-sectional study of 2,270 patient–caregiver dyads discharged from a tertiary ICU in Tianjin, China, to determine the prevalence and service-level structure of a high transitional-care gap across 15 services (primary outcome) and to assess whether that gap was associated with caregiving burden and psychological strain; residence-related differences in access barriers were examined as a secondary aim.

## 2 Methods

### 2.1 Study design and reporting

This single-center, cross-sectional study of patient–caregiver dyads after critical illness was conducted in the ICU at Tianjin First Central Hospital, China, between February 2022 and March 2026. The unit of analysis was the dyad: the family caregiver was the sole questionnaire respondent, and patient-level clinical variables were abstracted from the paired patient’s record. Reporting was structured according to the Strengthening the Reporting of Observational Studies in Epidemiology (STROBE) statement for cross-sectional studies (24). The study was designed to characterize caregiving burden, caregiver preparedness, psychological strain, patient vulnerability, and unmet transitional-care service needs, and to examine how these domains were distributed across clinical, sociodemographic, and rural–urban subgroups.

### 2.2 Setting and study period

Patient–caregiver dyads were recruited after hospital discharge from the ICU at Tianjin First Central Hospital, which serves both urban and rural catchment populations across the Tianjin region. Recruitment spanned three adult ICUs within the department—one medical, one surgical, and one mixed medical–surgical unit—with a total of 30 beds. Over the 50-month study period, the department recorded a mean of 1,200 ICU discharges per year, of which approximately 715 per year met the eligibility criteria in Section 2.3. The remainder were excluded primarily because of ICU mortality, stay shorter than 24 h, death before hospital discharge, absence of a family caregiving role, or diagnosed cognitive or communication impairment. The 2,270 dyads in the analytic sample were enrolled at a mean of 45 dyads per month and represent approximately 76% of eligible discharges during the recruitment window. Caregivers were assessed during a standardized early post-discharge window, when transitional care needs were expected to have emerged but recall decay was still limited. In the analytic cohort, assessment occurred a mean of 21.3 days after ICU discharge, with a standard deviation of 4.4 days. This window was selected to capture early PICS and family-caregiver strain during the initial transition from hospital-based care to home or community-based care, consistent with recommendations highlighting the importance of early post-ICU follow-up and recovery assessment (25, 26).

### 2.3 Participants and eligibility criteria

Eligible dyads included an adult patient aged 18 years or older who had been admitted to a medical, surgical, or mixed ICU for at least 24 h and had survived to hospital discharge, together with a self-identified primary family caregiver. The primary caregiver was defined as the unpaid family member who assumed principal responsibility for the patient’s post-discharge care, supervision, treatment coordination, or care-related decision-making. Only one caregiver was enrolled for each patient.

Caregivers were excluded if they were paid or professional carers, if they were unable to complete the structured questionnaire due to a diagnosed cognitive or communication impairment, or if the patient died or was readmitted to an ICU before the scheduled caregiver assessment. Patients whose post-discharge situation did not involve meaningful family caregiving were not approached. Patients requiring palliative or long-term-care planning could be included if a family caregiver remained responsible for decision-making or transitional-care coordination. Conversely, dyads were excluded if no family-caregiver was present at the time of assessment.

### 2.4 Sampling and recruitment

Every eligible dyad was invited in the order of ICU discharge throughout the recruitment period; none was selected based on availability. Sampling was therefore consecutive, a non-probability approach without a probability-based frame. Recruitment continued until the predefined recruitment period ended or the prespecified enrolment target was reached. The final analytic sample comprised 2,270 patient–caregiver dyads. Because the study was descriptive and hypothesis-generating, no single-hypothesis power calculation was performed. *Post hoc* assessment showed adequate precision at *n* = 2,270, with 95% CI half-widths of ±1.8% points for the primary outcome (75.3%) and ±1.0 point for low preparedness (5.8%). The four logistic models yielded approximately 16, 22, 3.8, and 16 events per variable across 35 candidate predictors, whereas linear models had approximately 82 observations per predictor. All models met conventional adequacy thresholds except the low-preparedness model, which may be overfitted and should be interpreted as exploratory.

### 2.5 Data collection procedures

Trained research assistants administered a structured caregiver questionnaire and abstracted patient clinical and discharge data from hospital records. Interviews were conducted in person during post-discharge contact or by telephone depending on caregiver preference and site logistics. Questionnaires were administered in Mandarin Chinese or, when required, in a locally appropriate dialect with support from trained study personnel. Data were entered into an electronic case-report form with predefined range checks, logical checks, and compulsory-field prompts for core variables. A random subset of records was checked against source questionnaires or interview records to reduce transcription error and improve data quality.

### 2.6 Exposure, outcome, and covariate framework

The primary outcome was the high transitional-care gap, defined as five or more unmet services across 15 transitional-care domains. Secondary outcomes included continuous caregiving burden, psychological strain, caregiver preparedness, patient vulnerability, unmet-needs count, access-barrier count, communication score, satisfaction score, and binary indicators of high caregiving burden, low preparedness, rehospitalization or emergency department attendance, and willingness to use digital follow-up. Patient clinical characteristics, caregiver sociodemographic characteristics, and caregiving-context variables were analyzed as covariates and candidate predictors. Rehospitalization or emergency department attendance was defined as any unplanned ward readmission or emergency visit after discharge and before caregiver assessment, based on caregiver report and, where available, hospital records. Patients readmitted directly to ICU before assessment were excluded (*n* = 143; 2.9% of screened ICU discharges) because transitional-care and caregiving measures lacked a stable post-discharge referent. Consequently, the reported outcome excludes the most severe deterioration and underestimates total unplanned healthcare utilization.

### 2.7 Patient and clinical variables

Patient-level variables abstracted from the medical record included age in years, sex, primary ICU admission diagnosis, ICU type, ICU length of stay, total hospital length of stay, receipt of invasive mechanical ventilation, duration of mechanical ventilation, delirium documented during the index ICU admission, tracheostomy at discharge, feeding tube at discharge, indwelling urinary catheter at discharge, pressure injury documented at discharge, and discharge disposition. Primary ICU diagnosis was grouped as respiratory failure, sepsis or shock, cardiovascular disease, neurological disease, trauma, post-operative admission, or other diagnosis. ICU type was classified as medical, surgical, or mixed ICU. Post-operative admission was defined as direct admission from the operating theatre for post-surgical monitoring, with trauma surgery classified separately. Because pre-operative counseling may influence caregiver preparedness, post-operative admission was included as a covariate in the adjusted low-preparedness model.

Functional status at discharge was recorded using a four-level ordinal scale comprising independent, some help required, most help required, and bedbound. For descriptive analyses and adjusted models, functional status was dichotomized as independent or some help versus most help or bedbound. Rehospitalization or emergency department attendance during the post-discharge assessment window was recorded by caregiver report and, where available, cross-checked against hospital records.

### 2.8 Caregiver sociodemographic and caregiving-context variables

Caregiver variables included age, sex, relationship to the patient, education, employment status, household income, residence, payment or insurance category, home-to-hospital distance, number of additional caregiving helpers, caregiving hours per day, caregiving days per week, and days since ICU discharge. Relationship to the patient was categorized as spouse, adult child, or other family caregiver. Education was dichotomized as college diploma or higher versus less than a college diploma. Employment status captured full-time employment before ICU admission, reduced hours or temporary leave, and resignation or job change after the patient’s critical illness.

Residence was classified as urban or rural using caregiver report and administrative classification. Home-to-hospital distance was dichotomized at 50 km or greater to represent a clinically relevant access barrier. Caregiving helpers were recorded as a count. Daily caregiving hours were collected using a five-level ordinal category and analyzed as an ordinal or approximately continuous caregiving-intensity variable. Caregiving days per week were recorded from 0 to 7.

### 2.9 Domain measures and score construction

Eight domains were derived from the caregiver assessment battery: caregiving burden, preparedness, communication, unmet needs, access barriers, patient vulnerability, psychological strain, and satisfaction. Caregiving burden, preparedness, patient vulnerability, and psychological strain were treated as reflective scales, for which Cronbach’s alpha was used to assess internal consistency. Unmet needs, access barriers, communication, and satisfaction were treated as formative indices comprising distinct, non-substitutable components; therefore, alpha was reported descriptively only (27). Measurement performance included score ranges, floor and ceiling frequencies, and internal consistency. The preparedness domain was informed by the Preparedness for Caregiving Scale construct but used study-specific items; therefore, its scores are not directly comparable to those of the published instrument. Internal consistency was modest (Cronbach’s *α* = 0.56), and preparedness findings were interpreted accordingly.

Caregiving burden was assessed using 10 items summed to a 0–40 score, with higher values indicating greater burden. Prespecified cut points defined high caregiving burden (≥26/40), low preparedness (≤2.00/4), high patient vulnerability (≥30/56), high psychological strain (≥28/40), low satisfaction (≤3.05/5), high transitional-care gap (≥5/15 unmet services), and high barrier burden (≥7/14 barriers). The latter corresponded to the upper tertile and classified 37.1% of participants because of tied discrete scores (28–30).

Preparedness was assessed using 12 items and expressed as a mean score from 0 to 4, with higher scores indicating greater readiness; responses marked not applicable were excluded from the respondent-level mean. Low preparedness was defined using the prespecified lower-score threshold. Communication was assessed using 15 items and summarized as a mean score from 1 to 5, reflecting the clarity, frequency, and usefulness of information received from clinical teams. Because the items represented distinct informational experiences, communication was treated as a multidimensional index.

Unmet transitional-care needs were assessed across 15 services, including clinical follow-up, medication review, rehabilitation, home nursing, device or wound training, nutritional and psychological support, social and financial support, palliative planning, emergency contact, and digital follow-up. Each service was classified as unmet when it was needed but was received inadequately or not at all. The unmet-needs score ranged from 0 to 15, and a high transitional-care gap was defined as ≥5 unmet services. The assessment framework was informed by established transitional-care and continuity-of-care models (31, 32). Access barriers were assessed using 14 checklist items covering structural, informational, financial, logistical, digital, family-related, and patient-related barriers. The barrier score was the number of endorsed items (0–14) and was treated as a formative count index because the barriers were conceptually heterogeneous.

Patient vulnerability was assessed using 14 items summed to a 0–56 score, with higher values indicating greater functional, clinical, and care-dependency vulnerability; high vulnerability was defined using the prespecified upper threshold. Psychological strain was assessed using 10 items summed to a 0–40 score, with higher values indicating greater caregiver emotional distress, anxiety, and worry; high strain was defined using the prespecified upper threshold (33). Satisfaction was assessed using 10 items, with a mean score ranging from 1 to 5, covering discharge preparation, communication, continuity, and the overall transitional-care experience. Because the items represented heterogeneous domains, satisfaction was treated as a multidimensional index; low satisfaction was defined using the prespecified lower threshold. Digital follow-up willingness was assessed using a single binary item indicating willingness to use WeChat- or telehealth-based follow-up after ICU discharge (34).

### 2.10 Statistical analysis

Analyses included all dyads with complete exposure-status classification (*n* = 2,270). Fully adjusted models used complete-case analysis (*n* = 2,227); multiple imputation was not performed because missingness for individual covariates was minimal. Continuous variables are summarized as mean ± standard deviation or median [interquartile range], and categorical variables as frequency (%). Characteristics were compared between caregivers with and without a high transitional-care gap using two-sample t-tests or Wilcoxon rank-sum tests, as appropriate, for continuous variables and chi-square tests for categorical variables. Standardized mean differences (SMDs) were also calculated, with |SMD| ≥ 0.10 considered a meaningful imbalance.

Measurement performance was summarized using observed score ranges, Cronbach’s alpha, distributional statistics, floor and ceiling frequencies, and the prevalence of prespecified risk categories. Cronbach’s alpha was interpreted for reflective scales only; for unmet needs, access barriers, communication, and satisfaction, it was reported descriptively because these domains were conceptualized as multidimensional indices (27). For each of the 15 transitional-care services, need was calculated using the full cohort as the denominator. Conversely, inadequate receipt, non-receipt, and total unmet need were calculated among participants reporting a need for that service. A non-receipt-to-unmet ratio was calculated to distinguish non-availability from inadequate service delivery.

Residence-related inequities were examined by comparing rural and urban caregivers across individual access barriers, total barrier score, high barrier burden, transitional-care gap, caregiving burden, preparedness, rehospitalization or emergency department attendance, and willingness to use digital follow-up. Risk differences were calculated as rural minus urban prevalence, and prevalence ratios as rural divided by urban prevalence, both with 95% CIs. Categorical outcomes were compared using chi-square tests and continuous barrier scores using two-sample t-tests. Because these residence analyses involved 21 comparisons, they were considered secondary, with *p* values between 0.01 and 0.05 interpreted as hypothesis-generating.

Spearman rank correlations were used to examine associations among clinical characteristics, caregiving intensity, and domain scores because several variables were ordinal or non-normally distributed. The 91 correlation coefficients were interpreted descriptively rather than as a confirmatory multiple-testing framework. Multivariable linear regression was used to examine factors associated with caregiving burden and psychological strain. Model assumptions were assessed using residual and partial-regression plots, Q–Q plots, Durbin–Watson statistics, variance inflation factors, and Cook’s distance >4/n. Because caregiving hours and burden were highly correlated (r = 0.93), the strain model was repeated after excluding caregiving hours.

Separate multivariable logistic regression models estimated adjusted odds ratios (aORs) with 95% CIs for high transitional-care gap, high caregiving burden, low preparedness, and rehospitalization or emergency department attendance. Outcome-defining variables were excluded from their respective models to avoid definitional circularity. Model discrimination and fit were assessed using the area under the receiver operating characteristic curve (AUC) and McFadden’s pseudo-R^2^, respectively. Models with limited event counts, particularly low preparedness, were considered exploratory. All tests were two-sided, with *p* < 0.05 considered statistically significant. Analyses were performed using R version 4.3.2.

## 3 Results

### 3.1 Participant flow and cohort characteristics

Of 5,004 ICU discharges screened over 50 months, 2,023 were excluded before eligibility assessment, primarily because of death before discharge (*n* = 806), ICU stay <24 h (*n* = 612), absence of an eligible caregiver (*n* = 318), direct ICU readmission (*n* = 143), death after discharge before assessment (*n* = 74), or caregiver cognitive or communication impairment (*n* = 70). Of 2,981 eligible dyads, 486 declined participation, and 225 were unreachable, yielding a final cohort of 2,270 dyads (76.1%). All participants contributed to descriptive analyses, while 2,227 (98.1%) with complete covariate data were included in adjusted models.

Patients had a mean age of 57.4 ± 14.4 years; 989 (43.6%) were women, and 1,483 (65.3%) received mechanical ventilation. The most common admission diagnoses were respiratory failure (28.2%), sepsis or shock (21.6%), cardiovascular disease (13.0%), and post-operative admission (13.0%). Caregivers had a mean age of 45.4 ± 11.8 years; 1,396 (61.5%) were women, 904 (39.8%) resided in rural areas, and 881 (38.8%) had a college education or higher. Assessment was completed 21.3 ± 4.4 days after ICU discharge. Table 1 presents baseline characteristics stratified by the primary outcome to assess covariate balance before adjustment.

**Table 1:** Cohort profile by transitional-care gap status.

Characteristic	No high gap (*n* = 560)	High gap (*n* = 1710)	SMD	*p*-value
Patient age, y	58.1 ± 14.3	57.2 ± 14.4	−0.1	0.222
Women	248 (44.3)	741 (43.3)	−0	0.693
Respiratory failure diagnosis	159 (28.4)	481 (28.1)	−0	0.904
Sepsis or shock diagnosis	110 (19.6)	380 (22.2)	0.06	0.198
Medical ICU	158 (28.2)	504 (29.5)	0.03	0.569
ICU length of stay, d	7.5 ± 6.7	7.0 ± 5.7	−0.1	0.098
Hospital length of stay, d	16.7 ± 9.3	16.2 ± 8.6	−0.1	0.236
Mechanical ventilation	364 (65.0)	1,119 (65.4)	0.01	0.85
Tracheostomy at discharge	63 (11.2)	224 (13.1)	0.06	0.253
Feeding tube at discharge	275 (49.1)	725 (42.4)	−0.1	0.006
Urinary catheter at discharge	314 (56.1)	930 (54.4)	−0	0.487
Pressure injury at discharge	83 (14.8)	314 (18.4)	0.1	0.056
Discharged home	245 (43.8)	716 (41.9)	−0	0.435
Functional status: most help/bedbound	255 (45.5)	831 (48.6)	0.06	0.208
Rehospitalization or ED visit	142 (25.4)	434 (25.4)	0	0.991
Caregiver age, y	45.2 ± 11.8	45.5 ± 11.8	0.03	0.584
Women	354 (63.2)	1,042 (60.9)	−0.1	0.336
Spouse caregiver	174 (31.1)	511 (29.9)	−0	0.595
Adult child caregiver	229 (40.9)	717 (41.9)	0.02	0.666
College diploma or higher	225 (40.2)	656 (38.4)	−0	0.444
Full-time employment before ICU	201 (35.9)	610 (35.7)	0	0.925
Reduced hours/temporary leave	247 (44.1)	786 (46.0)	0.04	0.444
Resigned or changed job	113 (20.2)	315 (18.4)	−0	0.356
Middle or higher household income	288 (51.4)	894 (52.3)	0.02	0.726
Rural residence	236 (42.1)	668 (39.1)	−0.1	0.196
Home-hospital distance ≥50 km	201 (35.9)	537 (31.4)	−0.1	0.049
Self-pay/commercial/other insurance	144 (25.7)	464 (27.1)	0.03	0.51
Caregiving helpers, n	1.2 ± 1.0	1.2 ± 1.0	0.05	0.294
Caregiving hours/day, ordinal	3.6 ± 1.1	3.4 ± 1.1	−0.1	0.008
Caregiving days/week	6.4 ± 0.8	6.4 ± 0.8	−0.1	0.105
Days since ICU discharge	21.3 ± 4.4	21.3 ± 4.4	0	0.979
Caregiving burden score	23.1 ± 7.8	22.1 ± 7.7	−0.1	0.008
Preparedness score	2.51 ± 0.38	2.56 ± 0.37	0.14	0.005
Communication score	3.22 ± 0.24	3.25 ± 0.25	0.11	0.023
Unmet need score	3.4 ± 0.8	6.5 ± 1.4	2.48	<0.001
Barrier score	5.8 ± 2.0	5.9 ± 1.9	0.05	0.343
Patient vulnerability score	28.2 ± 5.5	28.5 ± 5.3	0.05	0.289
Psychological strain score	27.8 ± 4.2	27.4 ± 4.1	−0.1	0.019
Satisfaction score	3.23 ± 0.28	3.24 ± 0.28	0.03	0.558
Willing to use digital follow-up	337 (60.2)	1,020 (59.6)	−0	0.824

Values are n (%) or mean ± SD. High transitional-care gap was defined as ≥5 unmet services and is the primary outcome; stratification displays covariate balance before adjustment. Comparisons are descriptive: at *n* = 2,270, differences below the prespecified |SMD| ≥ 0.10 threshold for meaningful imbalance may still reach *p* < 0.05. *p*-values compare high-gap and no-high-gap groups using chi-square tests for categorical variables and two-sample tests for continuous variables.

### 3.2 Measurement performance

**Figure 1:**
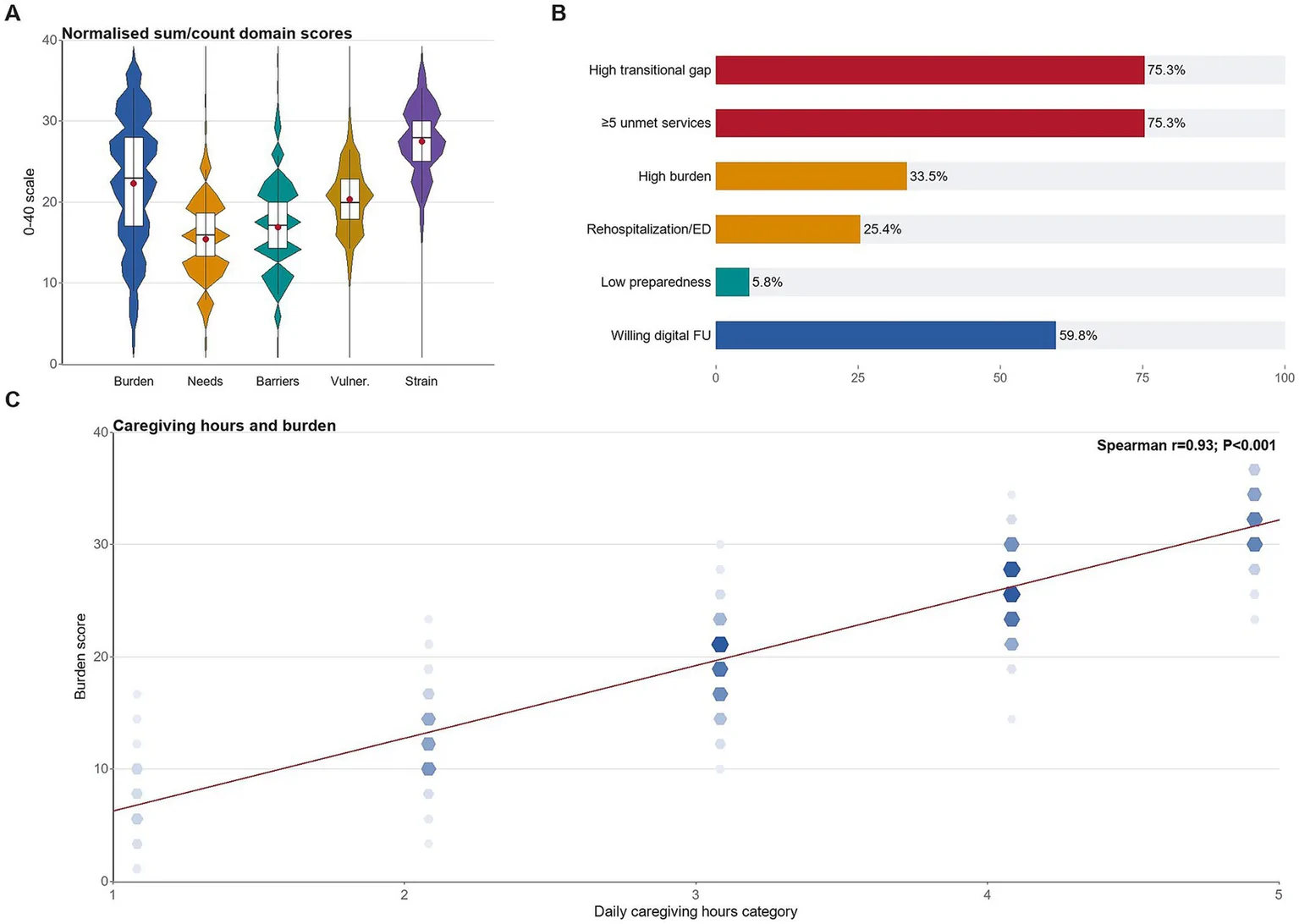
Post-ICU caregiver burden, outcomes, and caregiving intensity. **(A)** Distribution of five domain scores (caregiving burden, unmet needs, access barriers, patient vulnerability, and psychological strain), normalized to a common scale and shown as violin plots with embedded boxplots; points indicate group means. **(B)** Prevalence of five binary caregiver and patient outcomes across the analytic sample (*n* = 2,270): high transitional-care gap (defined as ≥5 unmet services of 15), high caregiving burden, rehospitalization/emergency department (ED) visit, low preparedness, and willingness to use digital follow-up. **(C)** Association between daily caregiving-hours category and caregiving burden score, with hexagonal binning reflecting point density and a fitted linear trend line.

Among the reflective scales, internal consistency was high for caregiving burden (*α* = 0.87) and modest for patient vulnerability and psychological strain (both α = 0.62). Preparedness showed lower internal consistency (*α* = 0.56) and was calculated from 1,935 respondents after excluding not applicable responses. As expected for formative indices, internal consistency was low for communication (*α* = 0.28), satisfaction (α = 0.12), access barriers (α = 0.10), and unmet needs (α = −0.04). Floor and ceiling effects were negligible across all domains (≤0.2%) (Table 2). The distributions of caregiving burden, unmet needs, access barriers, patient vulnerability, and psychological strain are shown in Figure 1A.

**Table 2:** Measurement performance and score distributions.

Domain	Items	Range	Alpha	Alpha n	Mean ± SD	Median [IQR]	Floor, *n* (%)	Ceiling, *n* (%)	Risk group, *n* (%)
Caregiving burden	10	0–40	0.87	2,270	22.3 ± 7.8	23 [17–28]	1 (0.0)	5 (0.2)	761 (33.5)
Preparedness	12	0–4	0.56	1,935	2.55 ± 0.37	2.56 [2.33–2.82]	1 (0.0)	1 (0.0)	132 (5.8)
Communication	15	1–5	0.28	2,270	3.24 ± 0.24	3.27 [3.07–3.40]	2 (0.1)	1 (0.0)	638 (28.1)
Unmet needs	15	0–15	−0.04	2,270	5.8 ± 1.8	6 [5–7]	1 (0.0)	1 (0.0)	1710 (75.3)
Access barriers	14	0–14	0.1	2,270	5.9 ± 1.9	6 [5–7]	1 (0.0)	1 (0.0)	843 (37.1)
Patient vulnerability	14	0–56	0.62	2,270	28.5 ± 5.3	28 [25–32]	1 (0.0)	2 (0.1)	808 (35.6)
Psychological strain	10	0–40	0.62	2,270	27.5 ± 4.1	28 [25–30]	1 (0.0)	1 (0.0)	963 (42.4)
Satisfaction	10	1–5	0.12	2,270	3.24 ± 0.28	3.20 [3.10–3.40]	2 (0.1)	1 (0.0)	552 (24.3)

Domain scores were calculated according to the codebook. Alpha denotes internal consistency. D6 responses coded not applicable were excluded from preparedness scoring. Floor and ceiling indicate observed minimum and maximum score frequencies. IQR = interquartile range; SD = standard deviation.

A high transitional-care gap was identified in 1,710 of 2,270 dyads (75.3%; 95% CI 73.5–77.1). High caregiving burden was present in 761 (33.5%), high psychological strain in 963 (42.4%), high patient vulnerability in 808 (35.6%), rehospitalization or emergency department visit in 576 (25.4%), and low preparedness in 132 (5.8%); 1,357 caregivers (59.8%) were willing to use digital follow-up (Figure 1B).

**Figure 2:**
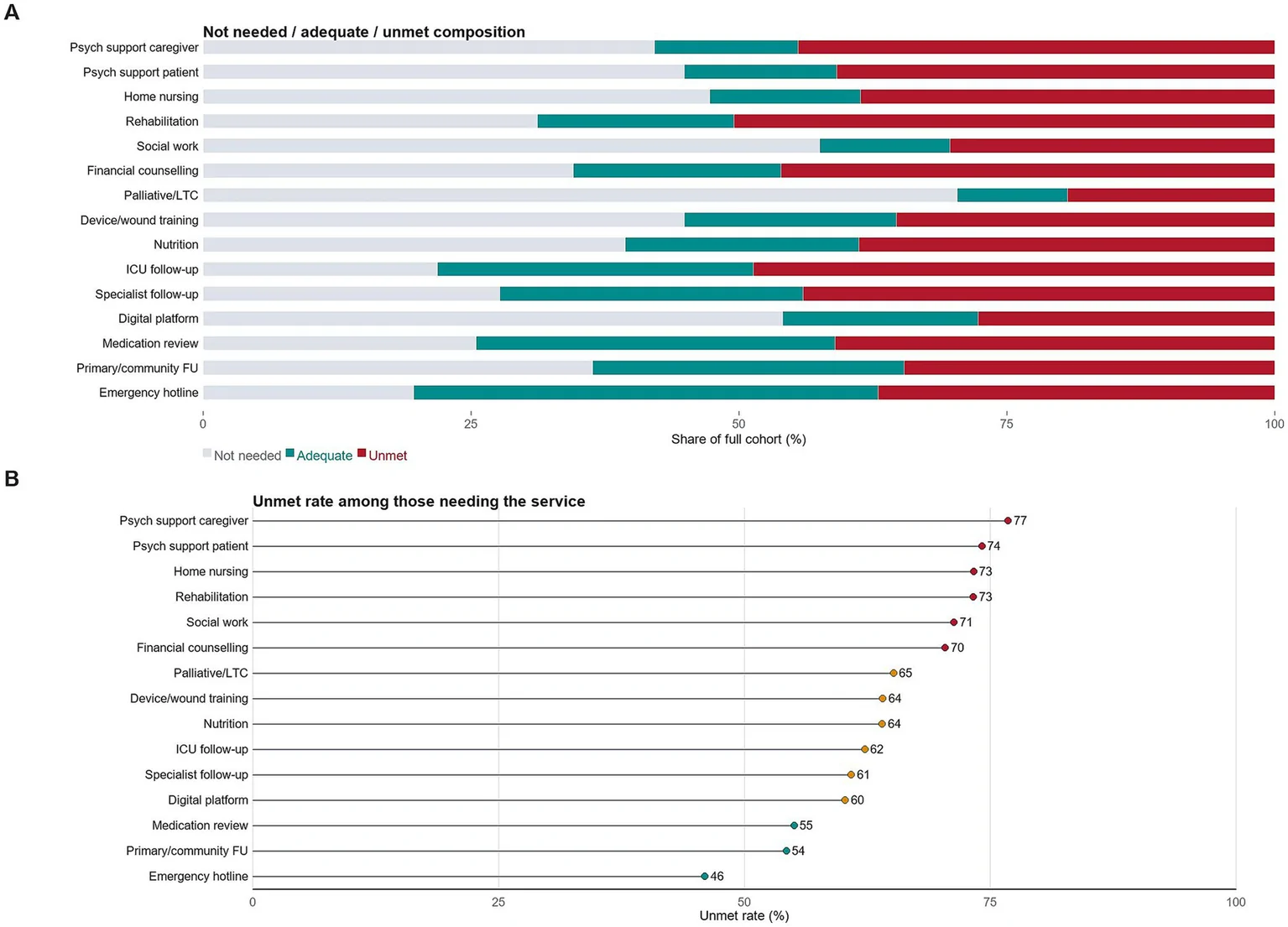
Transitional-care service needs and unmet care. **(A)** Composition of caregiver-reported need status across transitional-care services, classified as not needed, needed and adequately received, or needed and unmet (received inadequately or not received). **(B)** Unmet rate for each service, calculated as the proportion of caregivers who needed the service and had their need unmet, ranked from highest to lowest.

**Figure 3:**
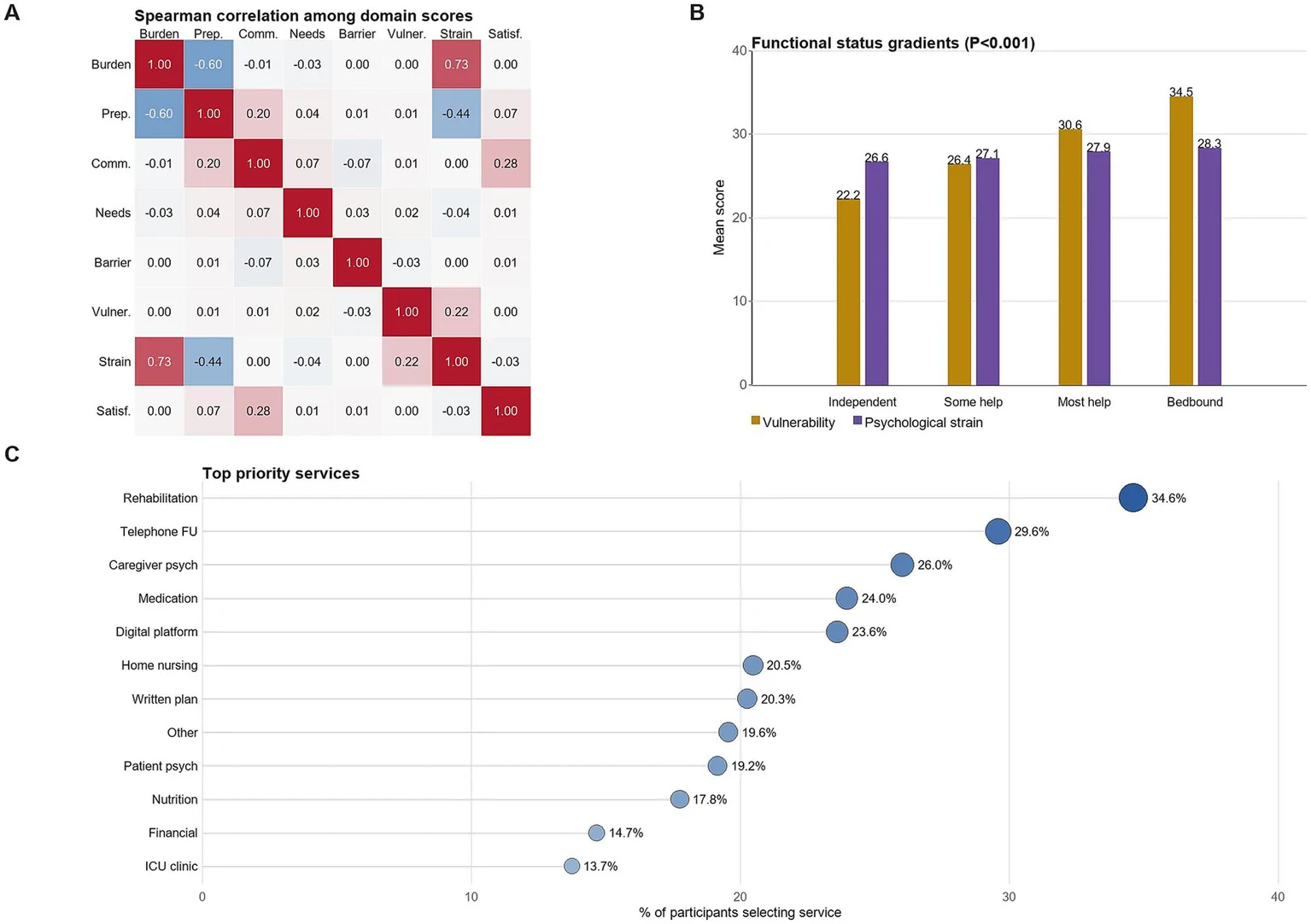
Correlation structure, functional status gradients, and service priorities: **(A)** Spearman correlation matrix among domain scores, with coefficients shown on a diverging color scale. **(B)** Mean patient vulnerability and psychological strain scores stratified by patient functional status at discharge. **(C)** Percentage of caregivers nominating each option as a top priority for improvement, ranked from highest to lowest. Priority options were a separate multiple-response item and do not correspond one-to-one with the 15 transitional-care services in Table 3; caregivers could select more than one, so percentages do not sum to 100.

The most frequently needed services were emergency contact or hotline access (80.4%), ICU physician follow-up (78.1%), and medication review (74.5%) (Table 3 and Figure 2A). Among caregivers reporting a need for each service, unmet need was highest for caregiver psychological support (76.8%), patient psychological counseling (74.2%), rehabilitation (73.3%), and home nursing (73.3%), and lowest for hotline access (46.0%) and primary or community follow-up (54.3%) (Figure 2B). Home nursing had the highest non-receipt-to-unmet ratio (0.66). Rehabilitation (34.6%), telephone follow-up (29.6%), and caregiver psychological support (26.0%) were most frequently identified as priorities for improvement (Figure 3C).

**Table 3:** Transitional-care need intensity by service domain.

Service	Needed, *n* (%)	Inadequate, *n* (% needed)	Not received, *n* (% needed)	Total unmet, *n* (% needed)	Nonreceipt/unmet ratio
Psychological support for caregiver	1,313 (57.8)	368 (28.0)	641 (48.8)	1,009 (76.8)	0.64
Psychological counseling for patient	1,250 (55.1)	373 (29.8)	554 (44.3)	927 (74.2)	0.6
Rehabilitation/physiotherapy	1,562 (68.8)	454 (29.1)	691 (44.2)	1,145 (73.3)	0.6
Home/community nursing visit	1,196 (52.7)	295 (24.7)	582 (48.7)	877 (73.3)	0.66
Social work support	963 (42.4)	283 (29.4)	404 (42.0)	687 (71.3)	0.59
Financial/insurance counseling	1,485 (65.4)	428 (28.8)	618 (41.6)	1,046 (70.4)	0.59
Palliative/LTC planning	672 (29.6)	183 (27.2)	255 (37.9)	438 (65.2)	0.58
Device/wound/tube training	1,250 (55.1)	370 (29.6)	431 (34.5)	801 (64.1)	0.54
Nutritional counselling	1,376 (60.6)	421 (30.6)	460 (33.4)	881 (64.0)	0.52
ICU/critical-care doctor follow-up	1773 (78.1)	513 (28.9)	591 (33.3)	1,104 (62.3)	0.54
Specialist follow-up	1,641 (72.3)	471 (28.7)	528 (32.2)	999 (60.9)	0.53
WeChat/digital support	1,042 (45.9)	334 (32.1)	294 (28.2)	628 (60.3)	0.47
Medication review	1,691 (74.5)	464 (27.4)	467 (27.6)	931 (55.1)	0.5
Primary/community follow-up	1,444 (63.6)	369 (25.6)	415 (28.7)	784 (54.3)	0.53
Emergency contact/hotline	1824 (80.4)	482 (26.4)	357 (19.6)	839 (46.0)	0.43

Need was defined as any response other than not needed. Unmet need included services received inadequately or not received. Needed percentages use *N* = 2,270; unmet percentages use the number needing each service. ICU = intensive care unit; LTC = long-term care.

### 3.3 Transitional-care gap, caregiving burden, and psychological strain

Clinical and demographic characteristics were generally well balanced between participants with and without a high transitional-care gap (all |SMD| < 0.10). Although seven characteristics reached nominal statistical significance, all standardized mean differences were ≤0.14, indicating small between-group differences. Unmet-need scores were substantially higher among participants with a high transitional-care gap (6.5 ± 1.4 vs. 3.4 ± 0.8, *p* < 0.001) (Table 1).

**Figure 4:**
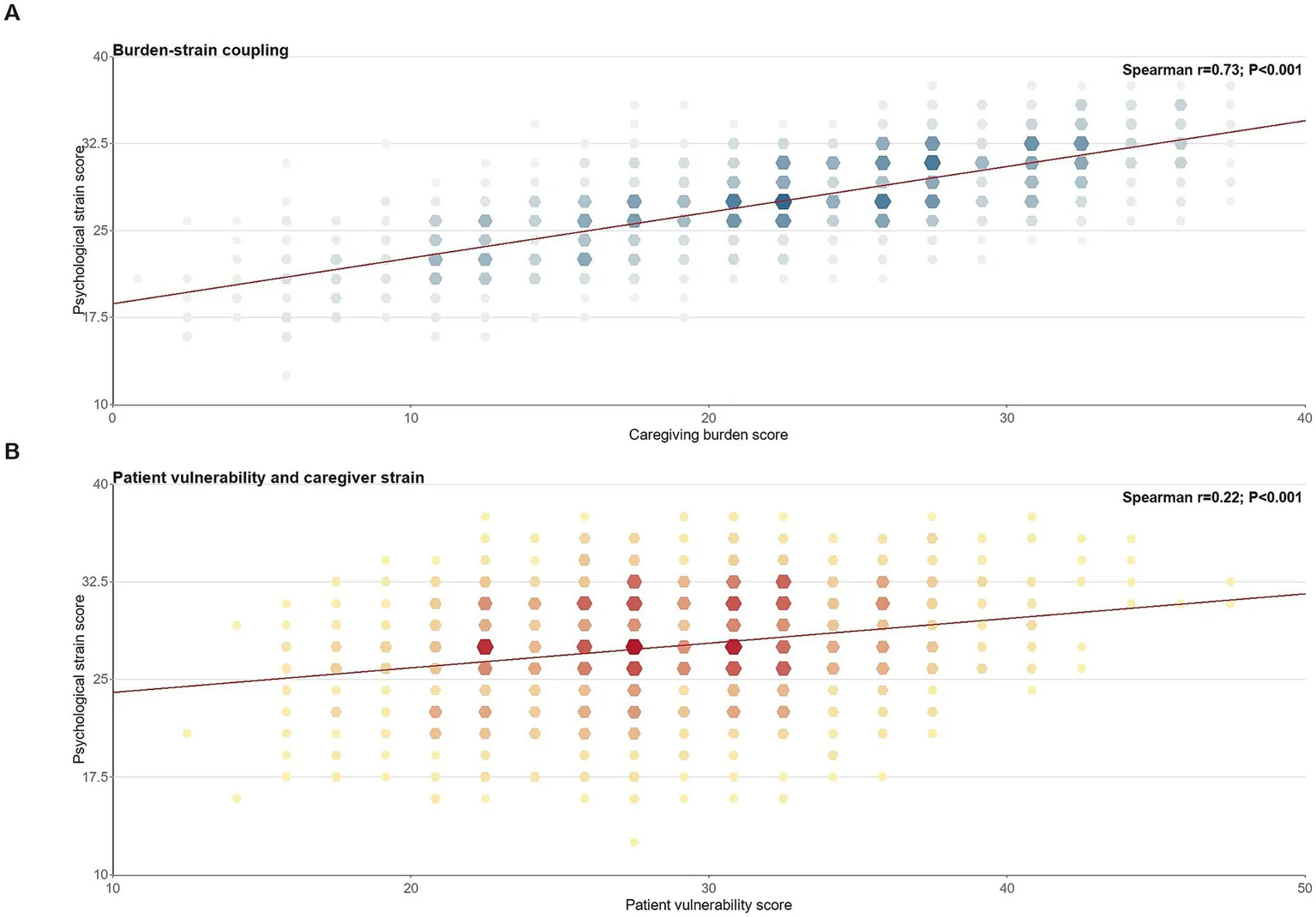
Coupled burden, vulnerability, and psychological strain. **(A)** Relationship between caregiving burden score and psychological strain score, shown as a hexagonal-binned scatter plot with a fitted linear regression line. **(B)** Relationship between the patient vulnerability score and psychological strain score, shown using the same format.

Unmet-needs and access-barrier scores showed little correlation with other domains (|*r*| ≤ 0.07). Conversely, caregiving hours were strongly correlated with burden (*r* = 0.93), burden with psychological strain (*r* = 0.73), and preparedness inversely with burden (*r* = −0.60) and strain (*r* = −0.44). Patient vulnerability showed weaker correlations with psychological strain (*r* = 0.22) and ICU length of stay (*r* = 0.23) (Table 4 and Figures 1C, 3A, 4A,B). After adjustment, unmet-need score was associated with neither caregiving burden (*β* = 0.00, 95% CI − 0.02 to 0.01, *p* = 0.69) nor psychological strain (*β* = −0.02, 95% CI − 0.04 to 0.01, *p* = 0.19); access-barrier score was similarly unrelated to either outcome (both *p* = 0.67) (Table 5).

**Table 4:** Spearman correlation matrix for clinical, caregiving, and domain variables.

Domain	Patient age	ICU LOS	Hospital LOS	Caregiver age	Distance	Hours/day	Burden	Preparedness	Communication	Unmet needs	Barriers	Vulnerability	Strain	Satisfaction
Patient age	—	0.03	−0.02	0	0.01	0.01	0	0	0.03	0.01	−0.03	0.01	0.01	−0.02
ICU LOS	0.03	—	0.64	0.02	0	0.03	0.02	0	−0.02	−0.03	−0.02	0.23	0.08	0
Hospital LOS	−0.02	0.64	—	0	0.03	0.01	0	0.01	−0.02	−0.02	−0.03	0.14	0.04	0.02
Caregiver age	0	0.02	0	—	0.05	0.02	0.01	−0.01	0.02	0	−0.01	0.01	0	0.03
Distance	0.01	0	0.03	0.05	—	−0.01	0	0.02	0	−0.02	0.03	0.02	0	−0.01
Hours/day	0.01	0.03	0.01	0.02	−0.01	—	0.93	−0.55	−0.01	−0.03	0	0	0.68	0
Burden	0	0.02	0	0.01	0	0.93	—	−0.6	−0.01	−0.03	0	0	0.73	0
Preparedness	0	0	0.01	−0.01	0.02	−0.55	−0.6	—	0.2	0.04	0.01	0.01	−0.44	0.07
Communication	0.03	−0.02	−0.02	0.02	0	−0.01	−0.01	0.2	—	0.07	−0.07	0.01	0	0.28
Unmet needs	0.01	−0.03	−0.02	0	−0.02	−0.03	−0.03	0.04	0.07	—	0.03	0.02	−0.04	0.01
Barriers	−0.03	−0.02	−0.03	−0.01	0.03	0	0	0.01	−0.07	0.03	—	−0.03	0	0.01
Vulnerability	0.01	0.23	0.14	0.01	0.02	0	0	0.01	0.01	0.02	−0.03	—	0.22	0
Strain	0.01	0.08	0.04	0	0	0.68	0.73	−0.44	0	−0.04	0	0.22	—	−0.03
Satisfaction	−0.02	0	0.02	0.03	−0.01	0	0	0.07	0.28	0.01	0.01	0	−0.03	—

Cells show Spearman rank correlation coefficients. Positive values indicate direct monotonic associations; negative values indicate inverse associations. ICU = intensive care unit; LOS = length of stay.

**Table 5:** Adjusted standardized linear models for caregiver burden and psychological strain.

Predictor	Burden *β*	95% CI	*p*-value	Strain β (95% CI)	*p*-value
Patient age	−0.01	−0.03 to 0.00	0.085	0.00 (−0.02 to 0.03)	0.848
ICU LOS	−0.01	−0.03 to 0.01	0.227	−0.01 (−0.04 to 0.03)	0.721
Hospital LOS	0.01	−0.01 to 0.03	0.396	0.01 (−0.02 to 0.05)	0.506
Mechanical ventilation	0.01	−0.01 to 0.02	0.391	0.00 (−0.02 to 0.03)	0.765
Delirium documented	−0.02	−0.03 to −0.00	0.043	−0.01 (−0.04 to 0.02)	0.423
Pressure injury	0	−0.01 to 0.02	0.940	−0.00 (−0.03 to 0.02)	0.857
Functional status	0	−0.02 to 0.02	0.971	−0.02 (−0.06 to 0.02)	0.391
Rehospitalization/ED	0	−0.02 to 0.01	0.593	−0.02 (−0.04 to 0.01)	0.235
Caregiver age	0	−0.02 to 0.01	0.678	−0.00 (−0.03 to 0.02)	0.920
Women	0	−0.02 to 0.01	0.910	0.01 (−0.02 to 0.03)	0.689
Education	0.08	0.06 to 0.10	<0.001	−0.02 (−0.06 to 0.01)	0.206
Employment change	0.01	−0.01 to 0.02	0.244	−0.02 (−0.05 to 0.00)	0.083
Income	0	−0.02 to 0.01	0.808	−0.03 (−0.05 to 0.00)	0.053
Rural residence	−0.01	−0.03 to 0.01	0.331	0.02 (−0.01 to 0.05)	0.144
Distance to hospital	0	−0.01 to 0.02	0.547	−0.00 (−0.03 to 0.02)	0.835
Helpers count	0.01	−0.01 to 0.02	0.487	−0.02 (−0.04 to 0.01)	0.236
Caregiving hours/d	0.83	0.81 to 0.85	<0.001	0.02 (−0.05 to 0.09)	0.601
Caregiving days/week	0	−0.01 to 0.02	0.720	−0.00 (−0.03 to 0.02)	0.885
Days since discharge	0.01	−0.01 to 0.02	0.445	−0.01 (−0.04 to 0.02)	0.452
Preparedness score	−0.18	−0.20 to −0.16	<0.001	0.02 (−0.02 to 0.06)	0.248
Communication score	0.01	−0.01 to 0.03	0.373	0.02 (−0.01 to 0.06)	0.15
Unmet need score	0	−0.02 to 0.01	0.691	−0.02 (−0.04 to 0.01)	0.186
Barrier score	0	−0.01 to 0.02	0.668	0.01 (−0.02 to 0.03)	0.668
Patient vulnerability	0.01	−0.02 to 0.03	0.553	0.25 (0.21 to 0.29)	<0.001
Satisfaction score	0	−0.02 to 0.01	0.532	−0.03 (−0.05 to 0.00)	0.066
Digital follow-up willingness	0	−0.02 to 0.01	0.613	0.01 (−0.02 to 0.03)	0.676
Burden score	—	—	—	0.73 (0.66 to 0.81)	<0.001
Model performance	Adjusted *R*^2^ = 0.876			Adjusted *R*^2^ = 0.608	

Values are standardized β coefficients from simultaneous-entry linear regression models. Models used complete cases after excluding unknown functional status (*n* = 2,227). Outcomes were caregiving burden and psychological strain. CI = confidence interval; ED = emergency department; ICU = intensive care unit; LOS = length of stay.

### 3.4 Factors associated with caregiving burden and psychological strain

The adjusted caregiving-burden model explained 87.6% of the variance (adjusted R^2^ = 0.876). Caregiving hours per day showed the strongest association with burden (*β* = 0.83, 95% CI 0.81–0.85, *p* < 0.001), whereas preparedness was inversely associated (β = −0.18, 95% CI − 0.20 to −0.16, *p* < 0.001) and education was positively associated (β = 0.08, 95% CI 0.06–0.10, *p* < 0.001).

**Figure 5:**
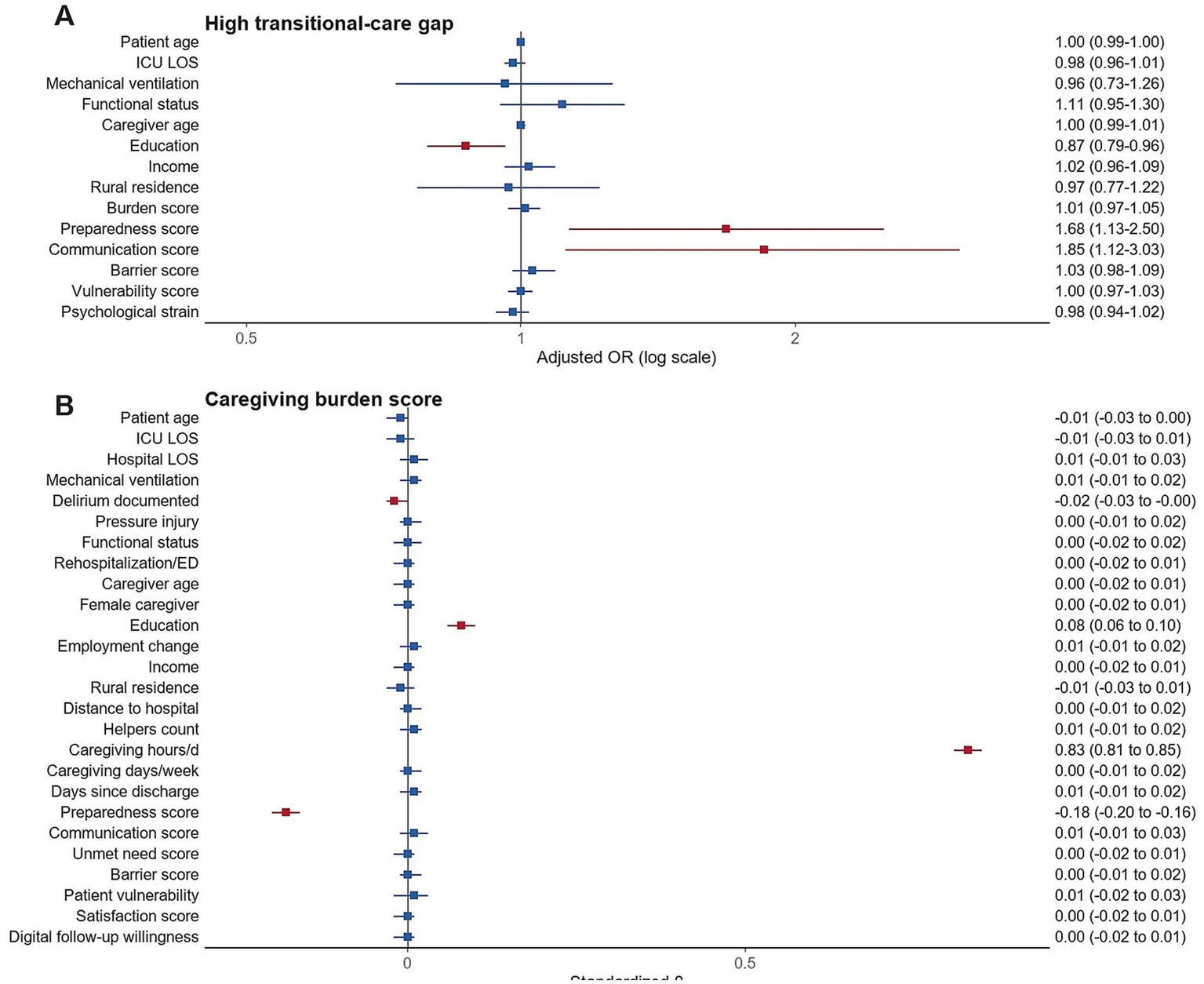
Adjusted models for transitional-care gap and caregiver burden. **(A)** Forest plot of adjusted odds ratios with confidence intervals from the multivariable logistic regression model for the high transitional-care gap; colored markers indicate statistically significant predictors. **(B)** Forest plot of standardized beta coefficients with confidence intervals from the multivariable linear regression model for the caregiving burden score; colored markers indicate statistically significant predictors.

The psychological-strain model explained 60.8% of the variance (adjusted *R*^2^ = 0.608). Caregiving burden (*β* = 0.73, 95% CI 0.66–0.81, *p* < 0.001) and patient vulnerability (*β* = 0.25, 95% CI 0.21–0.29, *p* < 0.001) were the strongest associated factors, whereas no sociodemographic variable was significant. Exclusion of caregiving hours produced nearly identical estimates and reduced the maximum variance inflation factor from 7.62 to 1.72 (Table 5 and Figure 5B). Functional dependency showed a graded association with vulnerability, increasing from 22.2 among independent patients to 34.5 among bedbound patients, and psychological strain, increasing from 26.6 to 28.3 (*p* < 0.001 for both) (Figure 3B).

### 3.5 Factors associated with the transitional-care gap and downstream outcomes

The adjusted model for a high transitional-care gap showed limited discrimination (AUC = 0.617; McFadden *R*^2^ = 0.028). Higher caregiver education was associated with lower odds of a high transitional-care gap (aOR 0.87, 95% CI 0.79–0.96, *p* = 0.003), whereas preparedness (aOR 1.68, 95% CI 1.13–2.50, *p* = 0.011), communication (aOR 1.85, 95% CI 1.12–3.03, *p* = 0.015), and pressure injury at discharge (aOR 1.45, *p* = 0.009) were associated with higher odds. Feeding tube at discharge (aOR 0.76, *p* = 0.007), greater distance to hospital (aOR 0.88, *p* = 0.009), and documented delirium (aOR 0.96, *p* = 0.040) were inversely associated (Table 6 and Figure 5A).

**Table 6:** Multi-outcome adjusted logistic model panel.

Predictor	High gap	High burden	Low preparedness	Rehospitalization/ED
Patient age	1.00 (0.99–1.00); *p* = 0.345	0.99 (0.98–1.00); *p* = 0.137	1.00 (0.99–1.02); *p* = 0.688	1.00 (1.00–1.01); *p* = 0.568
Women	1.02 (0.84–1.25); *p* = 0.843	0.83 (0.60–1.14); *p* = 0.252	1.37 (0.92–2.06); *p* = 0.123	0.96 (0.78–1.17); *p* = 0.660
Respiratory failure diagnosis	0.97 (0.92–1.03); *p* = 0.297	0.98 (0.90–1.08); *p* = 0.683	1.02 (0.91–1.14); *p* = 0.747	0.97 (0.92–1.02); *p* = 0.279
Medical ICU	1.00 (0.93–1.07); *p* = 0.945	1.06 (0.95–1.19); *p* = 0.307	1.04 (0.91–1.20); *p* = 0.574	1.02 (0.95–1.09); *p* = 0.540
ICU LOS	0.98 (0.96–1.01); *p* = 0.198	0.99 (0.95–1.03); *p* = 0.562	1.01 (0.96–1.06); *p* = 0.738	0.99 (0.96–1.01); *p* = 0.324
Hospital LOS	1.00 (0.99–1.02); *p* = 0.834	1.00 (0.98–1.02); *p* = 1.000	1.00 (0.97–1.03); *p* = 0.964	1.00 (0.99–1.02); *p* = 0.823
Mechanical ventilation	0.96 (0.73–1.26); *p* = 0.784	0.96 (0.62–1.48); *p* = 0.863	0.93 (0.53–1.63); *p* = 0.805	0.85 (0.65–1.11); *p* = 0.241
Ventilation duration	1.01 (0.96–1.05); *p* = 0.782	0.98 (0.92–1.05); *p* = 0.629	1.05 (0.97–1.14); *p* = 0.245	1.04 (1.00–1.09); *p* = 0.053
Delirium documented	0.96 (0.93–1.00); *p* = 0.040	0.96 (0.90–1.01); *p* = 0.136	0.96 (0.89–1.05); *p* = 0.375	0.98 (0.94–1.02); *p* = 0.267
Tracheostomy	1.21 (0.88–1.66); *p* = 0.233	0.83 (0.51–1.34); *p* = 0.440	0.75 (0.39–1.42); *p* = 0.373	0.89 (0.65–1.21); *p* = 0.449
Feeding tube	0.76 (0.62–0.93); *p* = 0.007	0.76 (0.55–1.05); *p* = 0.099	1.25 (0.83–1.88); *p* = 0.284	1.05 (0.86–1.27); *p* = 0.659
Urinary catheter	0.97 (0.80–1.19); *p* = 0.776	0.99 (0.72–1.37); *p* = 0.974	1.24 (0.82–1.89); *p* = 0.304	1.10 (0.90–1.34); *p* = 0.357
Pressure injury	1.45 (1.10–1.90); *p* = 0.009	1.05 (0.69–1.58); *p* = 0.827	0.96 (0.56–1.63); *p* = 0.875	0.85 (0.65–1.10); *p* = 0.220
Discharged home	1.06 (0.98–1.15); *p* = 0.159	0.98 (0.86–1.12); *p* = 0.790	0.91 (0.76–1.08); *p* = 0.269	0.92 (0.85–1.00); *p* = 0.046
Functional status	1.11 (0.95–1.30); *p* = 0.197	0.96 (0.74–1.23); *p* = 0.726	0.95 (0.68–1.34); *p* = 0.778	0.90 (0.77–1.06); *p* = 0.212
Caregiver age	1.00 (0.99–1.01); *p* = 0.587	1.00 (0.99–1.01); *p* = 0.812	1.00 (0.99–1.02); *p* = 0.840	1.00 (1.00–1.01); *p* = 0.255
Women	0.93 (0.75–1.13); *p* = 0.456	1.09 (0.78–1.52); *p* = 0.599	1.39 (0.91–2.13); *p* = 0.128	0.92 (0.75–1.12); *p* = 0.406
Adult child caregiver	1.03 (0.96–1.11); *p* = 0.364	1.02 (0.90–1.14); *p* = 0.785	1.07 (0.92–1.24); *p* = 0.397	1.07 (1.00–1.15); *p* = 0.060
Education	0.87 (0.79–0.96); *p* = 0.003	1.39 (1.20–1.62); *p* < 0.001	0.42 (0.34–0.51); *p* < 0.001	1.01 (0.92–1.10); *p* = 0.906
Employment change	0.96 (0.89–1.03); *p* = 0.273	1.13 (1.01–1.26); *p* = 0.038	1.04 (0.90–1.20); *p* = 0.624	1.08 (1.01–1.16); *p* = 0.030
Income	1.02 (0.96–1.09); *p* = 0.501	1.00 (0.91–1.11); *p* = 0.958	1.11 (0.98–1.26); *p* = 0.109	0.98 (0.92–1.04); *p* = 0.502
Rural residence	0.97 (0.77–1.22); *p* = 0.776	1.00 (0.69–1.45); *p* = 0.990	1.61 (1.00–2.58); *p* = 0.048	1.12 (0.90–1.41); *p* = 0.314
Distance to hospital	0.88 (0.80–0.97); *p* = 0.009	1.03 (0.88–1.19); *p* = 0.729	0.85 (0.70–1.04); *p* = 0.114	0.97 (0.89–1.07); *p* = 0.589
Helpers count	1.07 (0.96–1.18); *p* = 0.230	1.11 (0.94–1.30); *p* = 0.233	0.92 (0.74–1.13); *p* = 0.426	1.05 (0.95–1.17); *p* = 0.300
Caregiving hours/d	0.95 (0.75–1.21); *p* = 0.695	37.50 (22.87–61.47); *p* < 0.001	1.04 (0.60–1.79); *p* = 0.895	1.01 (0.80–1.28); *p* = 0.915
Caregiving days/week	0.90 (0.80–1.02); *p* = 0.107	1.00 (0.82–1.22); *p* = 0.990	1.09 (0.84–1.42); *p* = 0.506	1.10 (0.98–1.25); *p* = 0.116
Days since discharge	1.00 (0.98–1.02); *p* = 0.811	1.00 (0.97–1.04); *p* = 0.979	1.02 (0.97–1.06); *p* = 0.468	1.00 (0.98–1.02); *p* = 0.953
Burden score	1.01 (0.97–1.05); *p* = 0.518	Outcome scale	1.32 (1.21–1.44); *p* < 0.001	1.00 (0.96–1.04); *p* = 0.964
Preparedness score	1.68 (1.13–2.50); *p* = 0.011	0.08 (0.04–0.15); *p* < 0.001	Outcome scale	0.98 (0.66–1.45); *p* = 0.917
Communication score	1.85 (1.12–3.03); *p* = 0.015	1.13 (0.50–2.55); *p* = 0.777	1.16 (0.42–3.18); *p* = 0.774	1.20 (0.73–1.96); *p* = 0.473
Unmet need score	Outcome scale	0.99 (0.90–1.08); *p* = 0.751	0.98 (0.88–1.09); *p* = 0.652	1.00 (0.95–1.05); *p* = 0.968
Barrier score	1.03 (0.98–1.09); *p* = 0.236	0.97 (0.89–1.06); *p* = 0.556	1.05 (0.95–1.18); *p* = 0.342	0.97 (0.92–1.02); *p* = 0.226
Vulnerability score	1.00 (0.97–1.03); *p* = 0.816	0.98 (0.94–1.03); *p* = 0.469	1.02 (0.95–1.08); *p* = 0.632	1.06 (1.03–1.10); *p* < 0.001
Psychological strain	0.98 (0.94–1.02); *p* = 0.250	1.32 (1.24–1.40); *p* < 0.001	0.92 (0.85–1.00); *p* = 0.049	0.98 (0.94–1.01); *p* = 0.221
Satisfaction score	0.99 (0.69–1.43); *p* = 0.966	0.50 (0.27–0.93); *p* = 0.028	1.09 (0.51–2.34); *p* = 0.819	0.80 (0.55–1.14); *p* = 0.218
Digital follow-up willingness	1.08 (0.92–1.26); *p* = 0.369	0.92 (0.72–1.19); *p* = 0.547	1.07 (0.78–1.47); *p* = 0.661	1.08 (0.92–1.26); *p* = 0.353
Model performance	AUC = 0.617; R^2^ = 0.028	AUC = 0.964; R^2^ = 0.652	AUC = 0.896; R^2^ = 0.318	AUC = 0.601; R^2^ = 0.023

Cells show adjusted OR (95% CI); *p*-value from separate multivariable logistic regression models. Models used complete cases (*n* = 2,227). Reference categories are: men; surgical or mixed ICU; no mechanical ventilation; delirium not documented; no tracheostomy, feeding tube, or urinary catheter at discharge; no pressure injury; discharge other than home; functional status independent or some help (vs most help or bedbound); men; spouse caregiver (vs. adult child); less than college education; urban residence; and, for each admission diagnosis, all other diagnoses combined. Continuous predictors are per one unit: age in years; ICU and hospital length of stay, ventilation duration, and days since discharge in days; helpers as a count; caregiving hours/day per category on a five-level ordinal scale; caregiving days/week from 0 to 7; and domain scores per point on the ranges in Table 2. “Excluded (defines outcome)” marks a domain score omitted as a predictor from the model in which its own dichotomization is the outcome, avoiding definitional circularity: unmet-need score defines the high transitional-care gap (≥5 of 15 unmet services), burden score defines high caregiving burden (≥26 of 40), and preparedness score defines low preparedness (≤2.00 of 4). All four models otherwise included the same 35 candidate predictors; no cells are missing. AUC and McFadden *R*^2^ summarize model performance. The high-burden model is partly self-predictive because caregiving hours/days correlate with the burden score at *r* = 0.93, and its discrimination should not be read as an independent predictive pathway; the low-preparedness model is exploratory, with approximately 3.8 events per variable. AUC = area under the receiver operating characteristic curve; CI = confidence interval; ED = emergency department; ICU = intensive care unit; LOS = length of stay; OR = odds ratio.

For high caregiving burden, caregiving hours per day showed the strongest association (aOR 37.50, 95% CI 22.87–61.47, *p* < 0.001), whereas preparedness was protective (aOR 0.08, *p* < 0.001). The model showed high apparent discrimination (AUC = 0.964; McFadden *R*^2^ = 0.652), although caregiving hours were strongly correlated with the underlying burden score (*r* = 0.93). For low preparedness, education was protective (aOR 0.42, 95% CI 0.34–0.51, *p* < 0.001), while rural residence was associated with higher odds (aOR 1.61, 95% CI 1.00–2.58, *p* = 0.048). This model had approximately 3.8 events per variable and was therefore considered exploratory. For rehospitalization or emergency department visits, patient vulnerability was the strongest associated factor (aOR 1.06 per point, 95% CI 1.03–1.10, *p* < 0.001; 1.37 per SD), while discharge home was protective (aOR 0.92, *p* = 0.046) and caregiver employment change was associated with higher odds (aOR 1.08, *p* = 0.030).

### 3.6 Secondary analysis: residence and access barriers

**Figure 6:**
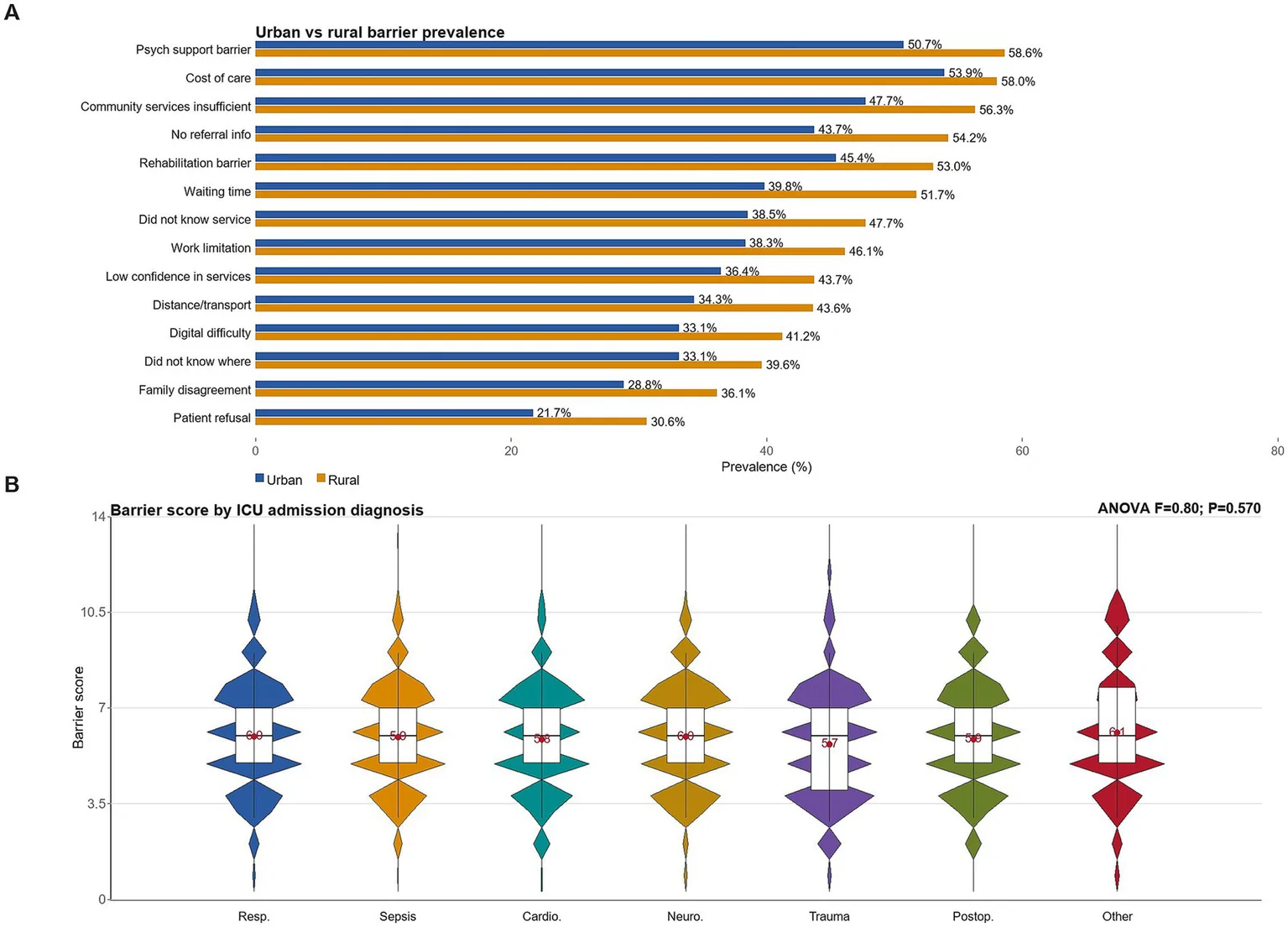
Barriers to transitional care by residence and diagnosis. **(A)** Prevalence of each of the 14 individual access barriers, compared between urban (*n* = 1,366) and rural (*n* = 904) caregivers, ranked by prevalence among rural caregivers. Thirteen differences were statistically significant; cost of care was not (58.0% vs. 53.9%, *p* = 0.055) and is shown with open bars. **(B)** Distribution of barrier score by intensive care unit (ICU) admission diagnosis category, presented as violin plots with embedded boxplots and group means.

Rural caregivers (*n* = 904) reported higher prevalence than urban caregivers (*n* = 1,366) for 13 of 14 access barriers. The largest absolute differences were observed for prolonged waiting time (51.7% vs. 39.8%; risk difference 11.8 percentage points, *p* < 0.001), lack of referral information (54.2% vs. 43.7%; 10.5 percentage points, *p* < 0.001), and distance or transport difficulty (43.6% vs. 34.3%; 9.3 percentage points, *p* < 0.001). Mean barrier scores were higher among rural caregivers (6.6 ± 1.9 vs. 5.5 ± 1.8, *p* < 0.001), and high barrier burden was nearly twice as prevalent (52.5% vs. 26.9%; prevalence ratio 1.95, *p* < 0.001) (Table 7 and Figure 6A).

**Table 7:** Residence-related inequity in access barriers.

Barrier	Urban (*n* = 1,366)	Rural (*n* = 904)	Risk difference, pp	Prevalence ratio	*p*-value
Waiting time too long	544 (39.8)	467 (51.7)	11.8	1.3	<0.001
No referral information	597 (43.7)	490 (54.2)	10.5	1.24	<0.001
Distance/transport difficulty	468 (34.3)	394 (43.6)	9.3	1.27	<0.001
Did not know which service was needed	526 (38.5)	431 (47.7)	9.2	1.24	<0.001
Patient refusal/unable to attend	296 (21.7)	277 (30.6)	9	1.41	<0.001
Community services insufficient	652 (47.7)	509 (56.3)	8.6	1.18	<0.001
Digital consultation difficult	452 (33.1)	372 (41.2)	8.1	1.24	<0.001
Psychological support barrier	692 (50.7)	530 (58.6)	8	1.16	<0.001
Work responsibilities limited capacity	523 (38.3)	417 (46.1)	7.8	1.2	<0.001
Rehabilitation unavailable/unaffordable	620 (45.4)	479 (53.0)	7.6	1.17	<0.001
Lacked confidence in available services	497 (36.4)	395 (43.7)	7.3	1.2	<0.001
Family disagreement	394 (28.8)	326 (36.1)	7.2	1.25	<0.001
Did not know where to obtain service	452 (33.1)	358 (39.6)	6.5	1.2	0.002
Cost of care	736 (53.9)	524 (58.0)	4.1	1.08	0.055
Barrier score, mean ± SD	5.5 ± 1.8	6.6 ± 1.9	1.1	—	<0.001
High barrier burden (upper tertile)	368 (26.9)	475 (52.5)	25.6	1.95	<0.001
High transitional-care gap	1,042 (76.3)	668 (73.9)	−2.4	0.97	0.196
High caregiving burden	456 (33.4)	305 (33.7)	0.4	1.01	0.86
Low preparedness	69 (5.1)	63 (7.0)	1.9	1.38	0.056
Rehospitalization or ED visit	341 (25.0)	235 (26.0)	1	1.04	0.58
Willing to use digital follow-up	819 (60.0)	538 (59.5)	−0.4	0.99	0.833

Residence was based on caregiver report. Risk difference is the rural minus urban prevalence in percentage points. Prevalence ratio is the rural prevalence divided by urban prevalence. *p*-values are from chi-square tests or two-sample tests. ED = emergency department; SD = standard deviation.

Despite these differences in access, rural and urban caregivers did not differ significantly in the prevalence of high transitional-care gap (73.9% vs. 76.3%, *p* = 0.196), high caregiving burden (33.7% vs. 33.4%, *p* = 0.86), low preparedness (7.0% vs. 5.1%, *p* = 0.056), rehospitalization or emergency department visit (26.0% vs. 25.0%, *p* = 0.58), or willingness to use digital follow-up (59.5% vs. 60.0%, *p* = 0.833). Barrier scores also did not differ across admission-diagnosis categories (*F* = 0.80, *p* = 0.570; Figure 6B).

### 3.7 Secondary analysis: preparedness by unit type and admitting diagnosis

Preparedness differed modestly by unit type, with higher scores in surgical or mixed units than in medical units (2.56 ± 0.37 vs. 2.52 ± 0.38; SMD 0.11, *p* = 0.019), while the prevalence of low preparedness was similar (5.3% vs. 6.9%, *p* = 0.150). More pronounced differences were observed by admitting diagnosis. Caregivers of post-operative patients had higher preparedness scores than those caring for patients with other diagnoses (2.63 ± 0.36 vs. 2.54 ± 0.37; SMD 0.25, *p* < 0.001), and low preparedness was less frequent (2.7% vs. 6.3%; prevalence ratio 0.43, *p* = 0.014). Communication scores were also higher among caregivers of post-operative patients (3.29 ± 0.24 vs. 3.23 ± 0.24, *p* < 0.001). In the adjusted low-preparedness model, post-operative admission remained independently protective (aOR 0.44, 95% CI 0.21–0.92, *p* = 0.029), and education remained protective (aOR 0.43, 95% CI 0.35–0.52, *p* < 0.001), whereas rural residence was no longer statistically significant (aOR 1.58, 95% CI 0.98–2.55, *p* = 0.061).

## 4 Discussion

In this cohort of 2,270 patient–caregiver dyads assessed after ICU discharge, unmet transitional-care needs were highly prevalent and concentrated in psychological support, rehabilitation, and home-based care. However, the magnitude of unmet need was largely independent of caregiver burden and psychological strain, which were instead closely related to caregiving intensity and patient dependency. A similar dissociation was evident geographically: rural caregivers reported substantially greater access barriers than urban caregivers, yet these barriers were not accompanied by greater transitional-care gaps, caregiver burden, psychological strain, or rehospitalization. Taken together, these findings suggest that the post-ICU transition encompasses at least two related but distinct challenges: inadequate availability or delivery of transitional-care services, shaped principally by health-system and geographic factors, and caregiver psychological burden, shaped more directly by caregiving intensity and residual patient vulnerability. Accordingly, service availability and caregiver distress should not be assumed to identify the same high-risk families and may require parallel assessment and intervention strategies.

Caregivers with and without a high transitional-care gap differed minimally in burden and psychological strain, with standardized mean differences ≤0.14. After adjustment, neither unmet-needs nor access-barrier scores remained associated with these outcomes. These findings should be interpreted as an absence of a clinically meaningful association rather than evidence of an inverse relationship. Nevertheless, this result differs from the conventional PICS-F framework, in which inadequate post-ICU support might be expected to exacerbate family distress. Several mechanisms may explain this apparent dissociation. First, ascertainment of unmet need depends on recognition of need: caregivers who were better prepared and communicated more effectively with clinical teams may have identified and requested a broader range of services, thereby increasing their measured unmet-needs count. Preparedness is a distinct and potentially modifiable caregiving construct rather than simply the inverse of burden (16, 17). Second, because approximately three-quarters of both rural and urban dyads met the threshold for a high transitional-care gap, limited variability at the upper end may have reduced the ability to discriminate among clinically distinct groups. Third, unmet needs and access barriers were treated as formative counts of heterogeneous and non-substitutable services, whereas burden and psychological strain were treated as reflective measures of underlying caregiver states. Their near-zero correlations may therefore reflect genuine construct separation rather than measurement failure. Finally, caregiver distress may emerge early during critical illness and remain strongly anchored to caregiving intensity and patient dependency after discharge, consistent with previous evidence linking caregiver burden more closely to caregiving demands than to service availability (35). Thus, a transitional-care gap score appears to characterize deficiencies in service provision more directly than caregiver psychological vulnerability, supporting separate screening for these domains.

The positive associations of preparedness and communication with a high transitional-care gap further support an ascertainment explanation. Caregivers who understood the patient’s post-discharge needs more clearly may have been better able to identify missing or inadequately delivered services, whereas less-prepared caregivers may have under-recognized such needs. This interpretation is consistent with validation work showing only a weak inverse correlation between preparedness and caregiver burden (Pearson *r* = −0.25) (22, 23). Conversely, preparedness and burden were more strongly inversely correlated in the present cohort (*r* = −0.60), suggesting greater overlap between readiness and caregiving experience in the early post-ICU period. Moreover, the very low internal consistency of the unmet-needs and access-barrier indices (*α* = −0.04 and α = 0.10, respectively), together with their negligible correlations with other domains, supports their interpretation as heterogeneous service indices rather than latent psychological scales (36, 37). This distinction is conceptually appropriate: a family may experience deficiencies in multiple services without necessarily experiencing a greater emotional burden, and conversely, substantial distress may occur despite adequate service provision.

Conversely, the relationships among caregiving intensity, burden, psychological strain, and patient vulnerability were internally consistent. Caregiving hours were strongly associated with burden, and burden with psychological strain. Patient vulnerability was independently associated with both psychological strain and rehospitalization. Graded increases in vulnerability and strain with worsening functional dependency further reinforce this pattern. Caregiver burden has previously demonstrated prognostic relevance rather than merely descriptive value; for example, each additional Zarit burden point was associated with higher odds of a 30-day emergency department revisit (19). Similarly, persistent disability is common among survivors of prolonged critical illness, with 64% retaining moderate-to-severe disability at 1 year in a previous cohort (21, 38). Such residual impairment may increase both caregiving demands and the likelihood of subsequent clinical deterioration, regardless of the availability of transitional-care services. These findings therefore position patient functional dependency as a clinically relevant upstream determinant linking post-ICU vulnerability to both caregiver strain and subsequent healthcare utilization (39, 40).

Education showed a more complex pattern. Higher caregiver education was associated with slightly greater burden but with lower odds of both a high transitional-care gap and low preparedness. These divergent associations argue against a simple causal interpretation of education as increasing caregiver burden. More highly educated caregivers may recognize caregiving demands more readily, articulate them more clearly, or hold higher expectations for post-discharge care, while also being better equipped to navigate healthcare services and prepare for caregiving responsibilities. Unmeasured correlates of education, including occupational commitments, availability of substitute caregivers, and socioeconomic circumstances, may also contribute. Consequently, education may influence both the recognition of caregiving demands and the capacity to navigate transitional care, rather than exerting a uniform effect on caregiver well-being.

The service-level findings identify clear priorities for improving transitional care. Unmet need was greatest for caregiver psychological support (76.8%), patient psychological counseling (74.2%), rehabilitation (73.3%), and home nursing (73.3%), whereas hotline access and primary or community follow-up were comparatively more available. This pattern suggests that resource-intensive services requiring specialist personnel or sustained face-to-face provision were less accessible than services that can be delivered remotely or through established outpatient pathways (53, 54). Psychological support remains particularly underrepresented in post-ICU intervention research, appearing in only six of 45 non-clinic-based interventions (41). When provided, such support has been associated with reductions in distress, anxiety, and depression that have persisted for up to 12 months among ICU families (12). The high prevalence of unmet psychological support needs in the present study therefore identifies this domain as a major target for transitional-care investment. Importantly, caregivers prioritized rehabilitation (34.6%), telephone follow-up (29.6%), and caregiver psychological support (26.0%), indicating substantial concordance between measured service deficits and caregiver-reported priorities.

Telephone- and technology-enabled approaches may offer a particularly scalable response. Nurse-led telephone follow-up has reduced 30-day readmissions (11, 42), and transitional-care interventions have improved symptom control in patients with COPD (10). Furthermore, 59.8% of caregivers in the present cohort expressed willingness to use digital follow-up, with no meaningful urban–rural difference. These findings align with growing evidence supporting technology-assisted post-discharge care (43, 44). A hybrid model combining structured telephone or digital follow-up with targeted referrals for rehabilitation and psychological care may therefore be more feasible than attempting to expand all components of transitional care uniformly, particularly in resource-constrained settings.

The rural–urban analysis further highlights the distinction between structural access and measured clinical or caregiver outcomes (55, 56). Rural caregivers reported higher prevalence of 13 of 14 barriers and nearly twice the prevalence of high barrier burden compared with urban caregivers (52.5% vs. 26.9%). The largest disparities involved waiting times, referral information, and transport, consistent with national evidence of lower healthcare capacity in economically disadvantaged and predominantly rural regions of China (14, 45). Nevertheless, these structural disadvantages were not associated with a greater transitional-care gap, caregiver burden, rehospitalization, or willingness to engage in digital follow-up. Moreover, the initial association between rural residence and low preparedness was attenuated after including post-operative admission and was driven by an underpowered model; rural residence should therefore not be regarded as an independent risk factor for the principal outcomes.

This apparent decoupling should not be taken as evidence that rural inequity is clinically unimportant. Extended family networks, informal caregiving arrangements, lower expectations of formal services, or other adaptive mechanisms may partially offset structural barriers in rural households (46, 47). Similar patterns have been observed in telemedicine research, where willingness to engage with remote care remained high despite substantial barriers to access and affordability (15). The absence of an urban–rural difference in willingness to use digital follow-up in the present cohort therefore suggests that remote transitional-care models could mitigate geographic barriers. However, whether digital care can translate willingness into equitable uptake and improved outcomes requires prospective evaluation, particularly because digital literacy, connectivity, and implementation capacity were not directly measured (48, 49). Preparedness also varied by the clinical context of ICU admission. Caregivers of post-operative patients reported higher preparedness and communication than caregivers of patients admitted for other conditions. Post-operative admission remained independently associated with lower odds of low preparedness. One plausible explanation is that anticipated surgical admissions offer opportunities for structured counseling and expectation setting before ICU care, whereas unplanned critical illness does not. Because elective and emergency surgery were not distinguished and pre-operative counseling was not directly measured, this explanation remains speculative. Nevertheless, the finding raises an important implementation question: whether elements of anticipatory communication routinely available around planned surgery could be adapted for families experiencing unplanned critical illness before hospital discharge.

The measurement findings also have methodological implications. Internal consistency varied by the conceptual structure of each domain: reflective measures showed higher consistency, whereas formative indices showed low alpha values, as expected when items represent distinct rather than interchangeable experiences. Similar heterogeneity across caregiver-related measures has been reported previously (50–52). Accordingly, low Cronbach’s alpha should not automatically be interpreted as poor measurement quality for formative indices such as access barriers or unmet service needs. Future studies should therefore explicitly distinguish between reflective scales and formative indices at the design and validation stages and select reliability criteria appropriate to the underlying measurement model.

This study has several limitations. First, the cross-sectional design precluded causal and temporal inference; all regression estimates therefore represent associations. The absence of an association between unmet need and caregiver burden does not establish that service deficiencies have no subsequent psychological consequences, particularly because both were assessed at a single time point. Longitudinal assessment beginning before hospital discharge and extending through the early recovery period would be required to determine whether service gaps precede, follow, or modify caregiver distress trajectories. Second, caregiving hours and burden were strongly correlated (*r* = 0.93). Although excluding caregiving hours from the psychological-strain model yielded stable estimates, the high-burden logistic model remained partially self-predictive, and its high discrimination should not be interpreted as evidence of an independent predictive pathway. Third, criterion validity was not established because no validated comparator instrument was administered concurrently. The preparedness domain was informed by, but did not reproduce, the Preparedness for Caregiving Scale and showed modest internal consistency (*α* = 0.56). Therefore, absolute preparedness scores should not be compared directly with those from the validated instrument. Test–retest reliability could likewise not be assessed from a single evaluation. Fourth, surgical admissions were not classified as elective or emergency, and pre-operative counseling was not recorded, preventing determination of whether anticipatory counseling explained the higher preparedness observed among post-operative caregivers. Fifth, assessment timing was indexed to ICU discharge rather than hospital discharge, so the actual period spent at home varied among participants and may have influenced the opportunity to encounter unmet needs and to utilize healthcare services. Moreover, patients who died before assessment, underwent direct ICU readmission, or lacked an eligible family caregiver were excluded. The resulting cohort therefore represents surviving, family-supported dyads and may underestimate the burden of adverse post-discharge outcomes among all ICU survivors. Caregiver-reported measures were also susceptible to recall and social desirability biases, although the relatively narrow assessment window, electronic consistency checks, and verification of a subset of records helped reduce measurement error to some extent. Finally, this was a single-center study using consecutive sampling, which limits external generalizability. Multiple comparisons were conducted without formal multiplicity adjustment, and the low-preparedness model contained approximately 3.8 events per variable. Accordingly, residence analyses should be regarded as secondary, correlations as descriptive, and associations with *p* values between 0.01 and 0.05 as hypothesis-generating. Multicenter longitudinal validation is therefore required before the identified thresholds, associations, or proposed care pathways are used for risk stratification or service planning.

## 5 Conclusion

In this study, among 2,270 dyads assessed 3 weeks after ICU discharge, a high transitional-care gap affected three-quarters of families and was concentrated in psychological support. It was not associated with caregiving burden or psychological strain, which instead were associated with caregiving intensity and patient functional dependency. Service availability and caregiver psychological load therefore represent separate problems: neither measure identifies families affected by the other, and each requires its own screening and intervention. Furthermore, rural caregivers reported more access barriers without correspondingly worse outcomes. Prospective multicenter studies using validated dyadic instruments are needed to establish temporal ordering.

## Data Availability

The raw data supporting the conclusions of this article will be made available by the authors, without undue reservation.
